# Microbiological and Physicochemical Profile of Traditionally Produced *Chouriça de Carne* Dry-Fermented Sausages: Towards Benchmarking of Products Against Established Quality Groups

**DOI:** 10.3390/foods13223705

**Published:** 2024-11-20

**Authors:** Ana Sofia Faria, Olga María Bonilla-Luque, Laís Carvalho, Nathália Fernandes, Miguel Angel Prieto, Vasco Cadavez, Ursula Gonzales-Barron

**Affiliations:** 1 Centro de Investigação de Montanha (CIMO), Instituto Politécnico de Bragança, Campus de Santa Apolónia, 5300-253 Bragança, Portugal; anafaria@ipb.pt (A.S.F.); laismagalhaescarvalho@hotmail.com (L.C.); nathaliaraquelx@gmail.com (N.F.); vcadavez@ipb.pt (V.C.); 2 Laboratório Associado para a Sustentabilidade e Tecnologia em Regiões de Montanha (SusTEC), Instituto Politécnico de Bragança, Campus de Santa Apolónia, 5300-253 Bragança, Portugal; 3 Universidade de Vigo, Nutrition and Bromatology Group, Department of Analytical Chemistry and Food Science, Instituto de Agroecoloxía e Alimentación (IAA)—CITEXVI, 36310 Vigo, Spain; mprieto@uvigo.es; 4 Department of Food Science and Technology, UIC Zoonosis y Enfermedades Emergentes (ENZOEM), CeiA3, Universidad de Córdoba, Campus Rabanales, 14014 Córdoba, Spain; v32boluo@uco.es

**Keywords:** dry fermented sausage, quality, safety, pathogens, proximate composition, principal component analysis, factor analysis, cluster analysis

## Abstract

The physicochemical and microbiological properties of traditional Portuguese ready-to-eat dry fermented sausage *chouriça de carne* samples from 14 regional producers were analysed and subjected to multivariate analysis to determine the relationships between them and to evaluate how the quality and safety of these sausages is affected by these properties. Producer-mean values for physicochemical analyses were quite variable, with intervals of 4.87–6.11 for pH, 0.803–0.965 for a_w_, moisture 19.5–48.5%, protein 32.0–60.1% (db), fat 22.0–53.3% (db), ash 3.52–9.69% (db), and carbohydrates 1.66–13.5% (db). Mesophilic counts varied (5.61–8.68 log CFU/g), while lactic acid bacteria were generally high (MRS: 8.21–10.2; M17: 7.66–10.0 log CFU/g). *S. aureus* was enumerated in levels up to 2.55 log CFU/g, while presumptive *C. perfringens* never surpassed 2 log CFU/g. *Salmonella* spp. and *Listeria* spp. were also detected in the samples tested. Principal component (PC) analysis yielded a three-dimension solution that explained 60% of the data variation; PC1 (26%) characterized chorizo formulations with more meat, while PC2 (19.3%) described sausages with longer/rapid fermentation, and PC3 (14.5%) highlighted *chouriços* with poorer hygiene. Cluster analysis identified three quality groups: (i) chorizos with high moisture, high protein content, and lowest pH; (ii) sausages with low moisture, high fat content, and elevated pH; and (iii) *chouriças* with high moisture and high protein but lower fat contents, low pH, and improved hygiene. Lastly, factor analysis yielded a varimax-rotated three-factor solution that explained 65% of the data, with similar results to PCA; factor 1 (23.5%) depicted chorizos with low pH but high moisture, factor 2 (20.8%) described sausages with more meat in the formulation, and factor 3 (20.6%) longer or rapid fermentation. Overall, the results evidenced the great variability in the quality attributes of Portuguese chorizo sausages, very likely to arise from multiple recipes, ingredients, and manufacturing practices. The definition of quality clusters is expected to play a crucial role for the self-denominated “artisanal” food companies to benchmark their *chouriço* sausages against the proper artisanal quality group.

## 1. Introduction

According to historical records, sausage production in Europe dates to Greek and Roman cultures as one of many ways to preserve meat after slaughter. Sausage-making remains a common practice throughout the Mediterranean region, and dry-cured sausages made primarily with pork products and with relatively long maturation periods prevail, often without an obvious distinction between the fermentation and drying stages, and generally without smoking. Most of these Mediterranean dry fermented sausages have a final pH ≤ 5.0–5.5, and their shelf life is mainly guaranteed by reduced water activity (<0.90–0.91), achieved through drying [1,2].

*Chouriça de carne*, also called *chouriço* or *linguiça de carne*, is a Portuguese ready-to-eat (RTE) dry-fermented sausage produced predominantly in the rural regions of Northern Portugal and Alentejo. It is made from diced pork meat and fat, seasoned with salt, garlic, bay leaf, and paprika or red pepper paste, and marinated in water and regional wine. After mixing, the batter is left to rest for up to four days at cool temperatures and then stuffed into natural pig casings and tied with the same thread on both ends, resulting in horseshoe-shaped sausages [3,4,5].

Traditionally, these sausages are produced without using lactic acid bacteria (LAB) starter cultures, or additives, but rather through spontaneous fermentation at moderate rates and cold temperatures by the naturally occurring LAB microbiota in the raw materials [5,6,7]. Unlike most other Mediterranean dry-fermented sausages, *chouriça de carne* is usually smoked, and in the case of sausages produced in the Trás-os-Montes region (Northern Portugal), smoking is intensive and uses regional woods for combustion, a process that, aside from adding to the development of flavour and colour in the *chouriça*, also contributes to its shelf life through the antimicrobial properties of its volatile compounds [4,5,8]. After smoking, drying continues, and acidification caused by LAB fermentation reduces the water activity, contributing to the safety of the final product [1,4].

In northern Portugal, *chouriça de carne* is a staple in the diets of rural populations, where its production and consumption have a long and rich history intimately linked to the nature of the geographical area and the culture and customs of its inhabitants. Eventually, what was a product exclusively for self-consumption became a source of economic income, produced mainly by artisanal manufacturers or small-scale industries, following old recipes or inspired by them, using processes that have not yet been much altered by technological innovation, attaining in some cases, the recognition of “traditional” or “artisanal” food product [4,9,10].

According to Portuguese legislation, artisanal activities and products “must be characterized by fidelity to traditional processes, in which personal intervention constitutes a predominant factor, and the final product’s manufacturing is individualized and genuine”. At the same time, the European Commission defines traditional food products as foodstuffs “that result from a mode of production, processing or composition corresponding to traditional practice or is produced from raw materials or ingredients that are those traditionally used” and by “traditional” it is understood that it has “proven usage on the domestic market for a period that allows transmission between generations” (at least 30 years) [11,12].

*Chouriça de carne* fits these definitions, and due to the vast number of small producers, the variability in recipes and in the quality of raw materials, low technological development, and unstandardised manufacturing processes, the resulting final products can present an extensive nutritional, organoleptic, physicochemical, and microbiological diversity [4,9,10]. To the consumer, this variability makes these products desirable, reinforcing the feeling of authenticity and craftsmanship of the artisanal products themselves. However, uncontrolled processes may lead to microbiological contamination and represent a health hazard to the consumer, breaking trust in artisanal/traditional products and causing economic losses for fragile local economies.

There are a great number of tests that can be used to assess the physicochemical and microbiological quality and safety of *chouriça de carne*, which are expected to be correlated due to biochemical and microbiological processes involved in the manufacture. Karlsson (1992) stated that the large number of measurements used to assess meat quality are usually related, and because of it, they can be replaced by fewer measurements without a significant loss of information [13]. This can be performed using multivariate analysis such as principal component analysis (PCA) or factor analysis. The reduction of the total number of variables to only two or three components is enough to explain the majority of the total variability of the original variables, allowing the identification of key factors contributing to the observed phenomena and ease in the recognition of patterns and clusters in a complex dataset.

The aim of this study was (i) to evaluate the variability of microbiological and physicochemical attributes of *chouriça de carne* commercialised by artisanal producers representative of northeastern Portugal; (ii) to understand the associations between the studied properties through quality maps generated by principal component analysis and factor analysis; and (iii) to determine the quality groups of *chouriça de carne* by k-means clustering analysis.

## 2. Materials and Methods

Finished *chouriça de carne* sausages were acquired from 14 producers located in the district of Bragança, northeastern Portugal, from the counties of Bragança, Carrazeda de Ansiães, Miranda do Douro, Mirandela, Mogadouro, Vila Flor, Vimioso, and Vinhais. Five sausages from the same production lot were purchased per producer at traditional fairs or butcher shops and then transported and stored in refrigeration at the laboratory until analysis. Producers were selected based on their commercialization of RTE *chouriça de carne*, the scale of production (regional/local small-scale producers), and origin from different geographic areas within the district.

Under aseptic conditions, the sausage casings were removed using sterilised instruments. Each sausage was cut into small pieces, with the contents divided for microbiological and physicochemical analyses.

### 2.1. Microbiological Analyses

Twenty-five grams of sausage were diluted in 225 mL sterile Buffered Peptone Water (Liofilchem, Roseto degli Abruzzi, Italy) and homogenised for 90 s (Interscience Bag Mixer 400, Saint Nom la Bretêche, France). Appropriate decimal dilutions were prepared in sterile Buffered Peptone Water (Liofilchem, Italy) for microbial enumeration.

For aerobic mesophilic counts, one mL volumes from appropriate decimal dilutions were incorporated into Plate Count Agar (Liofilchem, Italy) and incubated at 35 °C for 48 h [14]. For lactic acid bacteria (LAB) enumeration, one mL aliquots from sampling dilutions were incorporated into MRS and M17 agar (Liofilchem, Italy) (for the cultivation of *lactobacilli* and *lactococci*, respectively), overlaid with 10 mL of 1.2% agar (Liofilchem, Italy) and incubated at 30 °C for 48 h [15]. For enumeration of presumptive *Clostridium perfringens*, one mL volumes were incorporated into TSC Agar, supplemented with Egg Yolk Emulsion and D-Cycloserine (Liofilchem, Italy), and incubated at 37 °C for 48 h in anaerobic conditions [16]. For *Staphylococcus aureus* counts, 0.1 mL aliquots were spread-plated on Baird-Parker Agar supplemented with Egg Yolk Tellurite Emulsion (Liofilchem, Italy) and incubated at 37 °C for 48 h [17]. To enumerate *Listeria* spp., 0.3 mL volumes were spread onto Listeria Oxford Agar enriched with Listeria Oxford Supplement (Liofilchem, Italy) and incubated at 37 °C for 48 h [18]. The corresponding ISO norms were also applied to determine the presence of *Listeria* spp. and *Salmonella* spp. in 25 g of sausage [19,20]. Suspect *Listeria* spp. colonies were confirmed using a biochemical panel (Listeria System 18R kit, Liofilchem, Italy) and presumptive *Salmonella* spp. colonies were confirmed by serological agglutination test (Salmonella Latex Kit, Liofilchem, Italy). Plating was carried out in duplicate for all bacteria (except for *Listeria* spp. enumeration, plated in triplicate), and colony-forming units were transformed to log CFU/g.

### 2.2. Physicochemical Analyses

Physicochemical characterisation was carried out for the following parameters: pH, water activity (a_w_), and proximate composition (moisture, ash, protein, and fat).

For pH analysis, 10 g of chorizo were homogenised for 30 s (Interscience Bag Mixer 400, France) in 90 mL of deionized water, and the pH of the homogenate was determined in triplicate using a FiveGo pH meter F2 coupled with a LE438 IP67 probe (Mettler-Toledo, Greifensee, Switzerland). To quantify a_w_, portions of sausage were ground and pressed into disposable sample cups in duplicate, and the values were read in an Aqualab 4TE water activity meter (4TE Decagon, San Francisco, CA, USA). Moisture, ash, protein (Kjeldahl N × 6.25), and fat contents were determined according to ISO 1442:1997, ISO 936:1998, ISO 937:1978, and AOAC 960.39, respectively [21,22,23,24]. Ash, protein, and fat contents were expressed as a dry basis (db), and determinations were made in triplicate per sausage sample. Carbohydrates content (CHO) was estimated by deduction of the percentages of ash, protein, and fat (100% − %Ash (db) − %Protein (db) − %Fat (db)).

### 2.3. Statistical Analyses

The data on the microbiological and physicochemical properties obtained from the analysed chouriça samples (pH, a_w_, moisture, ash, fat, protein, CHO, counts of mesophiles, *S. aureus*, *Clostridium* spp., LAB on MRS, LAB on M17, detection of *Salmonella* spp., and detection and enumeration of *Listeria* spp.) were subjected to three types of multivariate analysis: (i) principal component analysis; (ii) factor analysis; and (iii) cluster analysis, with the purpose of (1) understanding any underlying interrelationship between variables; (2) reducing the dimensionality of redundant variables; (3) appreciating the variability of chouriça sausages within the studied properties’ maps; and (4) assessing the feasibility of establishing quality groups for this Portuguese food product. For each property, one-way analysis of variance (ANOVA) and Tukey’s test of mean comparisons among factories were conducted at alpha = 0.10 using the functions aov() and TukeyHSD() from the stats package in the R statistical software, version 4.4.2 [25].

#### 2.3.1. Principal Component Analysis

Principal component analysis (PCA) was conducted to assess the influence of the microbiological and physicochemical properties on the *chouriça* samples. A solution for three principal components was adopted to account for at least 60% of the total variance of the multivariate data. Subsequently, two bidimensional PCA maps representing all chorizo variables were generated by projecting the sample scores onto the principal components’ span. The function PCA() from the FactoMineR package and packages factoextra and corrplot were used in R statistical software [25].

#### 2.3.2. Factor Analysis

Factor analysis was performed using the function fa() from the psych package in the R statistical software [25].

#### 2.3.3. Cluster Analysis

Cluster analysis was carried out by k-means clustering using the function kmeans from the stats package and fviz_pca_biplot in the factoextra package of R statistical software [25]. The optimal number of clusters (k = 3) was determined by the elbow method and the gap statistic approach from the factoextra package.

## 3. Results and Discussion

The physicochemical and microbiological analyses presented in Table 1 revealed considerable variability, as demonstrated by the range of producer mean values registered for each Portuguese *chouriça de carne* property. Univariate ANOVA revealed significant differences amongst producers in all the studied properties, especially for protein, ash, pH, water activity, and moisture.

### 3.1. Physicochemical Properties of Chouriça de Carne

Protein (32.0–60.1% db) and fat (22.0–53.3% db) exhibited the greatest variability, a dispersion akin to that seen in traditionally produced Portuguese *Chouriça de Vinhais* [26] and *Chouriça de carne de Melgaço* [27], the latter showing a correlation between greater variation in protein and fat content and a more traditional type of manufacturing. This variability may also be related to the use of varying pork cuts and the manual cutting and stuffing associated with artisanal *chouriças*.

Ash content was likewise remarkably irregular amongst factories (3.52–9.69% db), implying the use of varying quantities of salt (NaCl) in the formulations, and probably the use of additives in the case of the highest ash values, but still in the range with other dry-fermented sausages like the Spanish *Chorizo Zamorano* (6.7–7.8% db) [28]. Protein and fat content values reflect the proportions of pork meat and fat used in formulation, the two main ingredients in *chouriça de carne*, while ash reflects the quantities of salts (NaCl and others) added to the batter [29].

For pH, producer-specific mean values ranged from 4.87 (95% Confidence Interval, CI: 4.81–4.93) to 6.11 (95% CI: 6.05–6.17), with most producers (11 of 14) registering pH mean values ≤ 5.5. Other studies have shown similar low acidity in Portuguese *chouriça*-like sausages, such as northeastern *chouriço* (pH = 5.37–5.82) [30], *linguiça* (pH = 5.36–5.51) [31], *salpicão* (pH = 5.38–6.12) [32], *Chouriça de Vinhais* (pH = 5.00–5.90) and *Salpicão de Vinhais* (pH = 5.10–5.60) [26], northwestern *Chouriça de carne de Melgaço* (pH = 5.00–5.54) [27], or *Catalão* from Alentejo (pH = 5.46–5.60) [33]. Other Iberian dry-cured sausages with equally low acidity are the Spanish *Galician Chorizo* (pH = 5.60–5.81) [29], the *Asturian Chorizo* (pH = 5.05–5.13) [34], or the *Chorizo Zamorano* (pH = 5.17–5.80) [28]. Thus, the pH of *chouriço de carne* compared well with other sausages that share a lengthy processing period.

Water activity also showed substantial variance (0.803–0.965), reflecting sausages’ moisture levels, which also differed greatly among producers (19.5–48.5%). Most factories (9 of 14) produced *chouriça* with a_w_ low enough to ensure the safety of the product (<0.91), similar to most Mediterranean dry-fermented sausages [28,29,34]. However, five manufacturers registered a_w_ mean values that exceeded 0.91. Although not unheard of [30,31,32,35], such high values indicate that dehydration was not successful or that the drying period was too short, which may pose a risk to microbiological safety, particularly if combined with ineffective acidification of the sausage [1].

### 3.2. Microbiological Properties of Chouriça de Carne

LAB were the dominant microflora in all samples, with MRS and M17 counts very often exceeding 8.0 log CFU/g, values on par with other dry-fermented sausages [30,33,34]. Only producers BRA 2 and MOG presented lower counts, between 7.5 and 8.0 log CFU/g (Table 1), while the highest LAB levels were registered for factory VF, surpassing 10 log CFU/g. LAB counts in MRS were generally higher than in M17, which, given the fact that MRS is used for culturing *Lactobacillus* species, while M17 is used for the growth of *Lactococcus* spp., seems to indicate a higher abundance of the first over the latter as the fermenting native flora.

Aerobic mesophilic bacteria counts ranged between 5.61 log CFU/g (95% CI 5.27–5.96) for producer MIR3 and 8.68 log CFU/g (95% CI 8.33–9.02) for enterprise VF, also in line with other dry-fermented sausages [30,33].

*S. aureus* was enumerated in samples from 10 producers with mean counts of 1.76–2.55 log CFU/g, and *Clostridium* spp. in sausages from 6 producers with mean counts of 0.759–1.848 log CFU/g (Table 1). *Salmonella* spp. was detected in one chorizo sample of producer VF and *Listeria* spp. in all five samples of producer DOU, but at levels below the limit of quantification of 1.2 log CFU/g.

### 3.3. Principal Component Analysis

**Figure 1:**
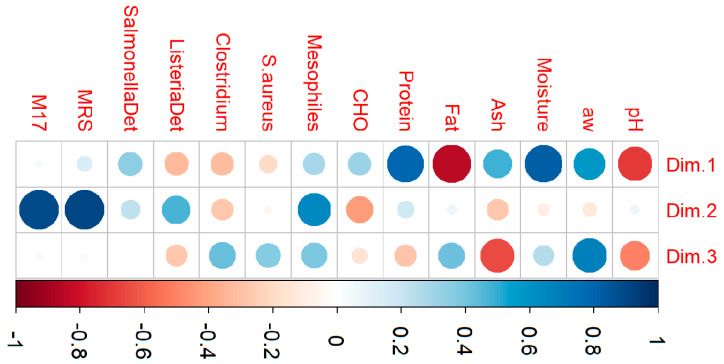
Representation of the coefficients of correlation obtained by principal component analysis for a solution of three dimensions accounting for 60% of the total data variability. The larger the circle, the higher the contribution of a variable to the dimension (Dim). Dark blue represents a highly positive correlation, whereas dark red represents a highly negative one; in any case, the lighter the colour, the weaker the correlation. Variables: pH; water activity (aw); moisture; ash; fat; protein carbohydrates (CHO); mesophiles count; *S. aureus* counts; *Clostridium* spp. counts; *Listeria* spp. detection (ListeriaDet); *Salmonella* spp. detection (SalmonellaDet); lactic acid bacteria count on MRS (MRS); lactic acid bacteria count on M17 agar (M17).

**Figure 2:**
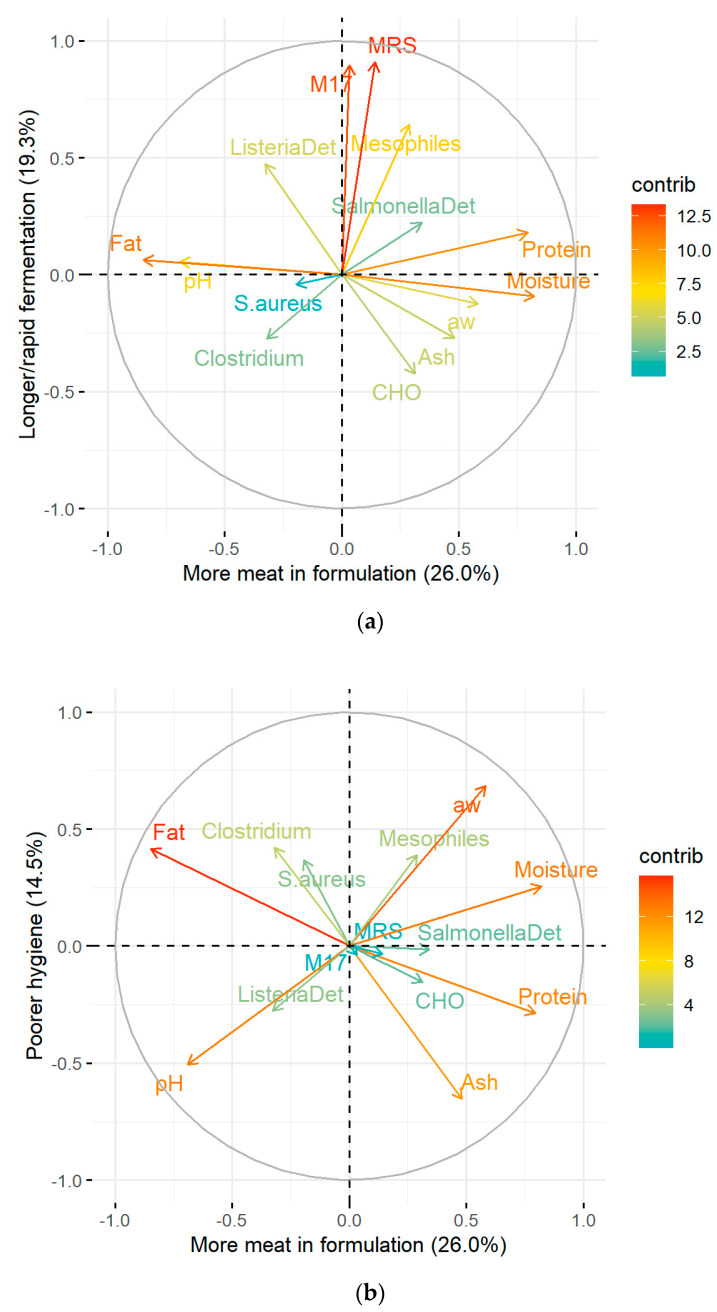
Variable correlation plots for the (**a**) first and second principal components, and the (**b**) first and third principal components of the compositional and microbiological attributes of Portuguese chorizo. An arrow represents the loading of a given variable, whereas its length/colour warmth indicates the extent of its contribution in projection to each of the components.

To understand how they impact the quality of the *chouriça*, PCA explored the relationship between the microbiological and physicochemical attributes. Since *Listeria* spp. was not enumerated, this variable was excluded from the analyses, and only the other 14 attributes were used. Supported by Kaiser’s criteria for eigenvalues (>1.0), the first three components were retained, with a cumulative variance of 60%. The coefficients of correlation obtained for the three-component solution can be seen in Figure 1, while the correlation plots for the principal components can be found in Figure 2.

PC1, the first and most important component, accounted for 26.0% of the variance registered. It was highly but negatively correlated to fat and pH, highly and positively associated with moisture and protein, and very moderately correlated to a_w_ and ash (Figure 1). The projection of variables in Figure 2a highlights the very close relationship between variables fat and pH, seen overlapping adjacent to the left side of the PC1 axis. Still, more importantly, the significant contribution in opposite directions of the axis of variables protein and fat indicates that PC1 was able to differentiate sausages with *more meat in its formulation* from those with more fat. This component was very much conditioned by product formulation, as pork meat and fat constitute the main ingredients of *chouriça de carne*. Patarata et al. observed a similar relationship between the variables for visual assessment of the amounts of meat and bread in the formulation of *alheira* sausages, as these constitute the essential ingredients of these sausages [36].

Additionally, alongside greater protein content, PC1 also described sausages marked by increased water content (high moisture and high a_w_) and low pH, which may be indicative of a shorter curing period and the use of additives. Dry-fermented sausages typically have low a_w_ (<0.9), one of the main hurdles preventing spoilage bacteria growth. However, to cut down production costs, some producers might resort to using acidification agents (such as lactic acid, in the form of its salt) to promote a rapid decrease in pH without having to sustain extended curing, that is, a long fermentation period (necessary for autochthonous flora development), in addition to drying. Furthermore, nitrates/nitrites (essential substrates for cured meat flavour and colour development) may be added to combat spore-producing anaerobic spoilage bacteria [37].

The correlation of ash to a_w_ and moisture may mean that this property indirectly indicates the use of additives and their impact on higher moisture retention. Ash is a measurement of all minerals and inorganic matter present in the sample after incineration at very high temperatures, including sodium chloride (NaCl) used in the formulation (and mostly responsible for the low a_w_) but also any additives [37].

Studies in Portuguese dry-fermented *chouriço*, *linguiça,* and *salpicão* revealed that factories using additives (polyphosphates, nitrates, and nitrites) in their formulations consistently produced sausages with higher a_w_ and moisture compared to factories that did not use any additives. This is due to shorter ripening periods and the polyphosphates’ capacity to increase the water-binding ability of fermented meats [30,31,32].

**Figure 3:**
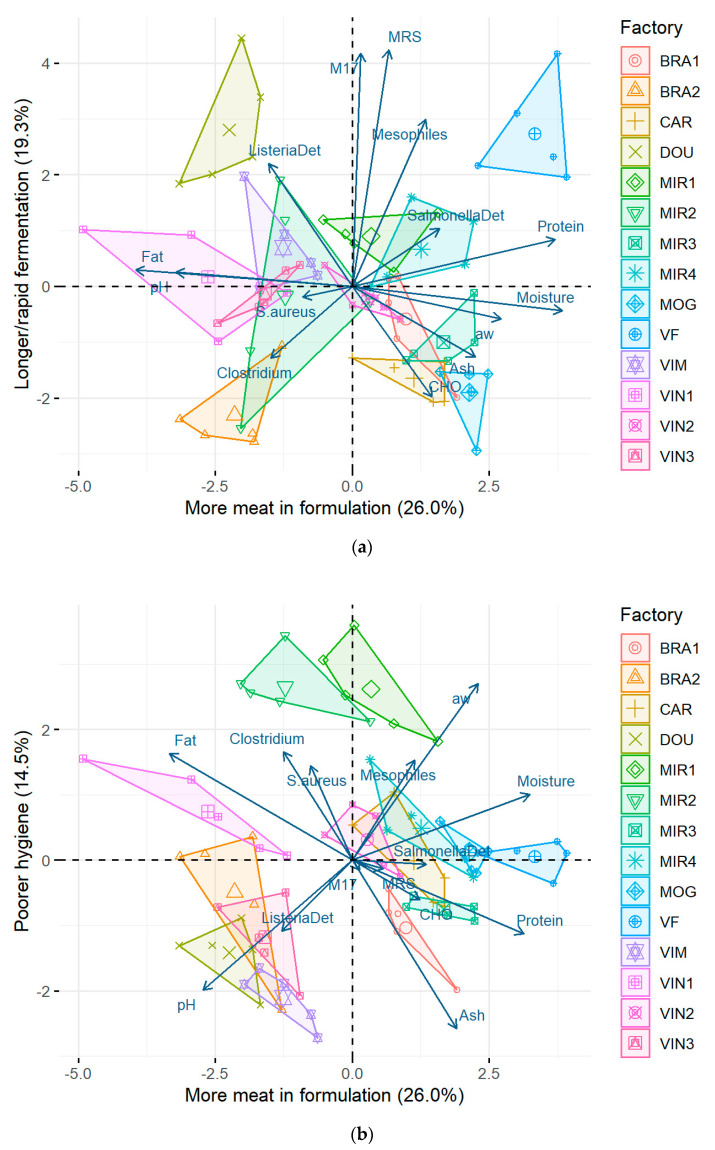
(**a**) First and second components, and (**b**) first and third components depicting a quality map of the compositional and microbiological attributes of Portuguese chorizo commercialised by 14 producers. An arrow represents the loading of a given variable, whereas its length indicates the extent of its contribution in projection to each of the components. Each colour and symbol represents a different producer. Whereas small symbols represent individual samples, large symbols are the centroid of the samples. Location of producers: BRA 1-2: Bragança 1-2; CAR: Carrazeda de Ansiães; DOU: Miranda do Douro; MIR 1-4: Mirandela 1-4; MOG: Mogadouro; VF: Vila Flor; VIM: Vimioso; VIN 1-3: Vinhais 1-3.

In the quality map shown in Figure 3a, it can be seen that producer VF employed the highest ratio of protein in its formulation, a high 60.1% on average, followed by producer MOG with 51.8%, according to Table 1, as opposed to factory VIN1, which used the least amount of protein in its sausages, with a mean of only 32.0% (Table 1). Unsurprisingly, VF and MOG were the enterprises that used the least fat in their formulations, with averages of 22.6% and 29.7%, respectively, and VIN1 used the highest content of fat, 53.3% on average (Table 1).

Factory MOG exhibited low pH (5.10) but high a_w_ (0.948) and high moisture content (48.5%), values aligned with a manufacturing process using a short curing period and artificial acidification using additives. This is corroborated by relatively low loads of LAB populations in the final product (MRS, 8.47 log CFU/g; M17, 7.66 log CFU/g) as well as the second-highest mean ash content registered across all producers (9.60%). In opposition, producer VF showed a lower pH (4.92) but also lower a_w_ (0.910) and moisture (40.1%), accompanied by high LAB concentrations (>10 log CFU/g) and lower ash content (7.83%), conditions consistent with a longer fermentation without resorting to preservatives.

The second component, PC2, justified 19.3% of the data and was highly correlated to lactic acid bacteria (from MRS and M17) and mesophilic bacteria (Figure 1). The high loadings and close relationships, particularly between MRS and M17, shown by their proximity on the upper right quadrant of the plot, near the PC2 axis, underlined the ability of PC2 to discriminate *chouriças* with *longer or faster fermentation* processes (Figure 2a).

During fermentation, lactic acid bacteria naturally present in raw meat materials become the dominant microflora. As they multiply, so does the production of lactic acid, the metabolic product of sugar glycolysis, causing the decrease of the pH of the sausage. This acidification is one of the main factors contributing to the inhibition of spoilage bacteria. Many factors affect the speed/length of the fermentation, namely the initial LAB loads naturally present on the raw materials, the addition or not of starter cultures, the temperature at which fermentation occurs (lower temperatures usually lead to a slower fermentation due to suboptimal growth conditions, resulting in a higher pH), or even the addition of sugars (glucose or lactose additives provide the ideal substrate for fermentation, accelerating LAB development and causing a more effective pH drop) [5,38].

In the quality map of Figure 3a, as discussed earlier, it is possible to see that enterprise VF benefited from a longer/faster fermentation in their chorizo production, mirrored by the high LAB counts in MRS (mean 10.2 log CFU/g) and M17 (mean 10.0 log CFU/g), followed by producer DOU (means of 9.88 log CFU/g on MRS and 9.44 log CFU/g on M17) (Table 1). On the opposite hand, factories BRA2 (MRS/M17: 7.76/7.86 log CFU/g) and MOG (MRS/M17: 8.47/7.66 log CFU/g) presented the sausages with the shortest/slowest fermentation (Table 1).

As for the third component, despite having a moderate weight, PC3 explained 14.5% of the data variance and was positively associated with water activity and, to a lesser extent, pathogenic agents presumptive *Clostridium perfringens* and *S. aureus*, and negatively correlated to ash (Figure 1). The projection of variables on the correlation plot revealed a close relationship between *Clostridium* spp. and *S. aureus*, seen in the upper left quadrant of the correlation plot (Figure 2b) near the axis of PC3, indicating this component’s capacity for identifying *sausages with poorer hygiene*, and with higher water activity as a potential driving factor for pathogen development, as seen by the high contribution of this variable to this component. On the contrary, ash, found directly opposite to the *Clostridium* spp. and *S. aureus* variables, may act as a pathogen-inhibiting factor and contribute to safer sausages.

As previously stated, ash measures all minerals and inorganic matter after heating at very high temperatures, including table salt (NaCl) and additives used for cured meat preservation. Despite current health concerns calling for the reduction of salt levels in cured meat products, traditional recipes of *chouriça de carne* still tend to use high levels to accomplish protein congealment, dehydration, and evaporation, causing a rapid reduction of the water levels during sausage curing, which hinders the development of spoilage and potentially pathogenic microorganisms.

Looking at the quality map of Figure 3b, factories MIR1 and MIR2 stand out as the enterprises with the poorest hygiene. According to Table 1, producer MIR1 had the second-highest mean counts of *S. aureus* (2.38 log CFU/g), while MIR2 presented the greatest mean counts of *Clostridium* spp. (1.848 log CFU/g). Regarding water activity, MIR1 registered the highest a_w_ mean value (0.965) and MIR2 the third most elevated one (0.934). As for ash, both exhibited the lowest mean values recorded, MIR2 with 3.52% ash (db) and MIR1 with 4.51% (db).

These values seem to indicate that curing was not efficient; the drying period was likely not long enough, resulting in insufficient water loss and elevated final water activity, an open pathway for the growth of pathogenic and spoilage bacterial populations, as seen by the elevated counts of *Clostridium* spp. and *S. aureus*, when compared to other producers. Reduced salt concentrations in these sausages’ formulations may also have contributed to inefficient curing, as highlighted by the low ash values registered compared to the rest of the producers. Typically, salt is added to dry-fermented sausage batter at concentrations of around 2.5–3% [4,39], resulting in concentrations that can reach over 4% in the end products [26,27,28,35,40], which would be reflected in a slightly higher ash content as well [27,28]. NaCl at levels lower than 2.5% would be insufficient for an effective reduction in the final a_w_ of *chouriça*, a necessary hurdle against the development of spoilage and pathogenic bacteria [39]. The low ash concentrations also suggest that no other additives were used in the formulation of these sausages.

Lastly, three of the studied attributes—ListeriaDet, SalmonellaDet, and CHO—did not generate any particularly high or moderate loadings in any of the three dimensions. This means that the detection of *Listeria* spp. and *Salmonella* spp. are random events, whereas CHO is a residual calculation, and any of these variables are in any way correlated to greater meat content, longer/faster fermentation, or poorer hygiene of the sausages either.

### 3.4. Cluster Analysis

The quality grouping of *chouriças* according to the principal components extracted was solved by non-hierarchical k-means clustering. Due to their low loadings, variables ListeriaDet and SalmonellaDet were removed from the analysis, and three clusters were identified.

**Figure 4:**
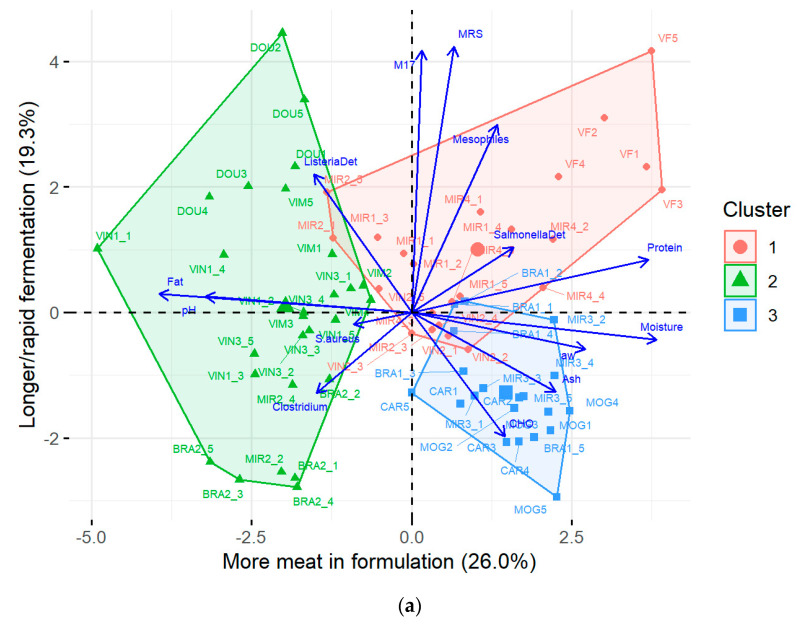

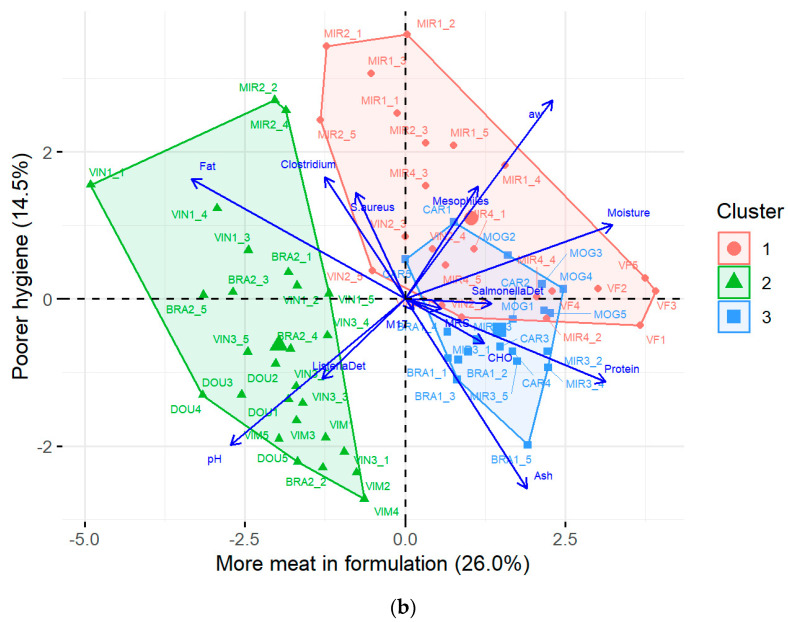
Quality grouping of Portuguese chorizo projected on the (**a**) first and second components and (**b**) first and third components, as solved by the k-means clustering algorithm (k = 3). An arrow represents the loading of a given variable, whereas its length indicates the extent of its contribution in projection to each of the components. Each colour represents a different cluster. Smaller symbols represent the projection of individual samples, whereas the larger symbols are the centroids.

Cluster 1 grouped chorizos with the lowest pH, high water content, and high protein content (Table 2), and according to the quality maps was composed by producers MIR1, MIR2 (3 of 5 sausages), MIR4, VF, and VIN2 (Figure 4).

Cluster 2 characterized chorizos with opposite characteristics to cluster 1; cluster 2 grouped sausages with elevated pH, low water content, and high fat content (Table 2), and included factories BRA2, DOU, MIR2 (two of five sausages), VIM, VIN1, and VIN3 (Figure 4).

As for cluster 3, it grouped *chouriças* with properties like those in cluster 1 (low pH, high water content, and high in protein) but also lower fat contents, high ash levels, and improved hygiene due to lower *S. aureus* and *Clostridium* spp. counts (Table 2), and comprised producers BRA1, CAR, MIR3, and MOG (Figure 4).

Cluster 1 seems to characterize more industrialized *chouriças*, with some type of starter culture added to the batter, as evidenced by its high LAB counts. This allows a faster pH drop and yields more acidic and moister sausages since they do not experience such extensive curing to achieve a low pH.

On the other hand, cluster 2 seems to represent rather traditionally prepared *chouriças*. The very low water content (a_w_ and moisture) is typical of long-cured artisanal sausages, whereas intermediate LAB population counts are characteristic of fermentation by the native flora. Another feature very typical of artisanal sausages is the high-fat content, as these sausages used to be part of a farmer’s diet and meant to be highly caloric, to provide a good source of energy for a hard day of labour in the cold winter months of the Trás-os-Montes region.

Lastly, cluster 3 appears to illustrate the most industrialized *chouriças*, as seen by low pH and high moisture/a_w_, together with high ash, low LAB counts, and low *S. aureus*/*Clostridium* spp. counts. Low pH was likely not achieved by native LAB fermentation, given the average counts registered, but probably through the addition of acidifying agents (e.g., lactate salts), later reflected in the high ash levels. High moisture/a_w_ also indicates reduced curing time, which seriously impacts the microbiological safety of the *chouriças*. Preservatives, specifically nitrates/nitrites, which prevent the development of *Clostridium perfringens* as seen by the lowest mean counts registered for this pathogen among the three clusters, would also increase ash levels.

### 3.5. Factor Analysis

Given that PCA yielded a three-component solution, three factors were also extracted from the factor analysis. CHO, ListeriaDet, and SalmonellaDet variables were also removed from factor analysis for their low loadings.

**Figure 5:**
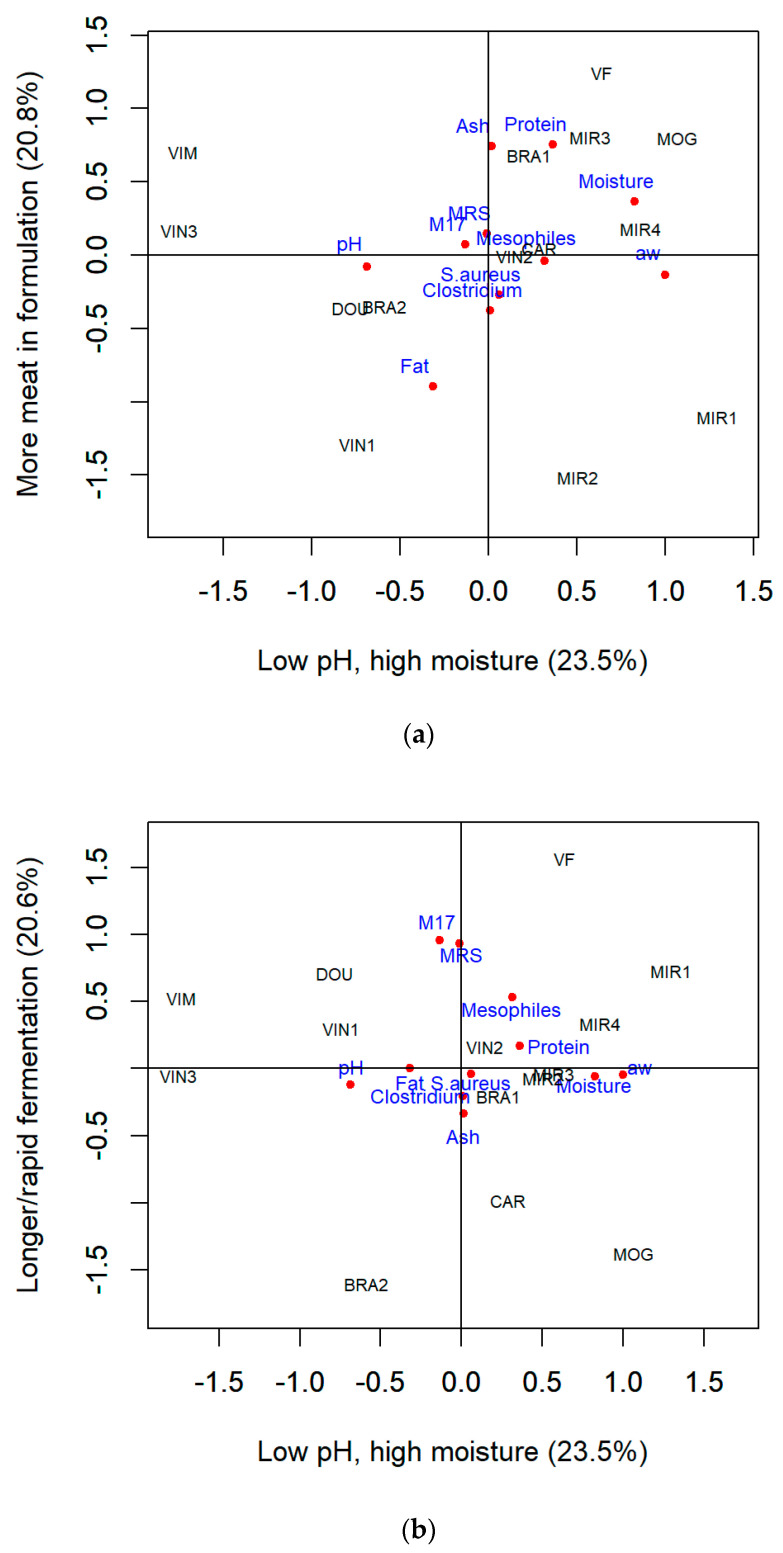
(**a**) First and second factors and (**b**) first and third factors of a varimax-rotated three-factor analysis solution displaying loadings of the compositional and microbiological attributes of Portuguese chorizo and the centroids of the factory scores. Each red dot represents the loading of a studied variable. The shorter the distance between a variable label and an axis, the greater its contribution to the dimension represented in such axis.

Despite being built on a separate multivariate algorithm, factor analysis results were similar to PCA. Factor analysis presented only marginally stronger relationships between variables since it explained a slightly higher total variability, 64.9%. The first factor justified 23.5% of the data variance and was highly correlated to water activity and moisture (R = 0.99 and R = 0.83, respectively) (Table 3). Additionally, factor 1 was negatively associated with pH (R = −0.69), as seen by the projection of the variable in the opposite direction of the factor 1 axis (Figure 5a), which together with the two moisture-associated variables’ loadings, indicates that this factor characterizes chorizos with *low pH and high moisture*, a latent dimension in the PCA although these same variables presented strong to moderate loadings in its first component.

Factories VIM and VIN3 produced the driest chorizos with the highest pH, while producers MOG and MIR1 presented the lowest pH and highest moisture (Figure 5a). Achieving an efficient drop in pH without loss of moisture in traditionally manufactured dry-fermented sausages is quite difficult, so some of these producers may likely use additives, such as acidifying agents, preservatives (nitrites/nitrates), phosphates, and maybe even starter cultures, to promote rapid acidification and prevent the development of undesirable microorganisms, while still obtaining *chouriças* with high moisture retention levels. This would allow the factory to shorten the curing period instead of relying on prolonged fermentation and drying processes typical of naturally fermented sausages.

Factor 2 explained 20.8% of the data variability and was highly but negatively associated with fat (R = −0.90) and positively correlated with protein and ash (R = 0.76 and R = 0.74, respectively) (Table 3). The projection of variables in Figure 5a demonstrates a close relationship between ash and protein, seen along the factor 2 axis on the right upper quadrant of the plot; together with the disposition in the opposite direction of the fat variable, it only consolidates the observation that factor 2 can identify *chouriças with more meat in its formulation*, as opposed to fattier ones, similar to principal component 1 identified through PCA.

According to Figure 5a, producer VF presented the sausages with the highest protein content (and lowest fat). Instead, sausages from producer VIN1 had the highest fat content (and lowest protein), as observed in the PCA analysis for PC1 (Figure 3a).

Lastly, factor 3 (20.6% of the data variance) was highly correlated to lactic acid bacteria (from M17 and MRS, R= 0.96 and R = 0.93, respectively) (Table 3). The high loadings and close relationships between M17 and MRS, shown near overlapping on the upper left quadrant of the plot, adjacent to the axis of factor 3, underlines the ability of this factor to differentiate *sausages with longer or rapid fermentation* times (Figure 5b), which matches component 2 detected by PCA. According to the plot in Figure 5b, VF is the enterprise with longer/faster fermentation, and factories BRA2 and MOG presented the sausages with the shortest/slowest fermentation, the same enterprises detected by PCA according to PC2 (Figure 3b).

## 4. Conclusions

*Chouriça de carne* is a Portuguese dry-fermented traditional sausage that has been poorly studied and characterised regarding its microbiological quality and safety. Overall, the results presented great chemical, physical, and microbiological variability, highlighting the difference in recipes, ingredients, and manufacturing practices among enterprises, contributing to a rich landscape.

Multivariate analysis identified protein-to-fat ratio, the extent of fermentation and the pH and water content as the main drivers of variability in these sausages, while simultaneously allowing for discrimination between *chouriças* linked to a more artisanal manufacturing process from those produced on a more industrial scale.

Group 2 of the cluster analysis, which presumably corresponds to “real” artisanal *chouriça de carne*, was characterized by higher pH, lower water content, and higher fat. In opposition, more industrialized *chouriças* were marked by lower pH and higher water contents. The results of group 2 may be useful for regional producers to benchmark their products against, to increase their commercial value and desirability in the market and in the eyes of the consumer.

Lastly, the variability between lots of the same industry is responsible for the unpredictable quality and safety of these RTE sausages. Regarding microbiological safety, the identification of several different pathogenic or potentially pathogenic microorganisms in these sausages—*Salmonella* spp., *Listeria* spp., *S. aureus*, *Clostridium* spp.—also stresses the importance of implementing adequate protocols to ensure the microbiological control of ingredients, the sanitization of all equipment/utensils, and the standardization of manufacturing processes, but also to reinforce good hygiene practices alongside the operators, to reduce the introduction of pathogenic agents and to strengthen the hurdles that avoid their development.

## Figures and Tables

**Table 1:** Compositional and microbiological attributes of Portuguese chorizo by sampled factory. Mean and 95% confidence interval are shown. For every attribute, different superscript letters indicate significant differences at α = 0.10.

Factory	Protein (% db)	Fat (% db)	Carbohydrates (% db)	Ash (% db)
BRA1	51.8 ^B^ [47.5–56.1]	30.7 ^C^ [25.5–35.9]	7.85 ^B^ [5.48–10.2]	9.69 ^A^ [9.03–10.4]
BRA2	40.0 ^D^ [35.6–44.2]	42.8 ^B^ [37.6–48.0]	9.36 ^B^ [7.00–11.7]	7.91 ^B^ [7.25–8.56]
CAR	44.2 ^C^ [39.9–48.5]	36.4 ^BC^ [31.2–41.7]	9.66 ^B^ [7.29–12.0]	9.66 ^A^ [9.00–10.3]
DOU	45.7 ^C^ [41.4–50.0]	45.3 ^B^ [40.1–50.5]	1.66 ^C^ [0.00–4.03]	7.32 ^B^ [6.66–7.98]
MIR1	40.5 ^D^ [36.3–44.8]	47.1 ^B^ [41.8–52.3]	7.88 ^B^ [5.52–10.3]	4.51 ^DE^ [3.85–5.17]
MIR2	44.9 ^C^ [40.7–49.2]	47.4 ^B^ [42.5–52.9]	3.85 ^C^ [1.48–6.21]	3.52 ^E^ [2.86–4.17]
MIR3	54.1 ^B^ [49.8–58.4]	26.9 ^CD^ [21.7–32.2]	11.2 ^AB^ [8.85–13.6]	7.79 ^B^ [7.13–8.45]
MIR4	54.1 ^B^ [49.8–58.3]	31.9 ^C^ [26.7–37.2]	7.26 ^BC^ [4.90–9.63]	6.74 ^C^ [6.08–7.40]
MOG	51.8 ^B^ [47.5–56.1]	29.7 ^C^ [24.5–34.9]	8.94 ^B^ [6.58–11.3]	9.60 ^A^ [8.94–10.3]
VF	60.1 ^A^ [55.8–64.4]	22.6 ^D^ [17.3–27.8]	9.49 ^B^ [7.13–11.9]	7.83 ^B^ [7.18–8.49]
VIM	47.9 ^B^ [43.6–52.1]	36.8 ^BC^ [31.5–42.0]	7.51 ^BC^ [5.14–9.87]	7.87 ^B^ [7.21–8.53]
VIN1	32.0 ^E^ [27.7–36.3]	53.3 ^A^ [48.1–58.6]	9.69 ^B^ [7.33–12.1]	4.98 ^D^ [4.33–5.64]
VIN2	50.5 ^B^ [46.2–54.8]	30.7 ^C^ [25.5–35.9]	13.5 ^A^ [11.1–15.8]	5.36 ^D^ [4.70–6.02]
VIN3	42.8 ^C^ [38.5–47.1]	40.6 ^B^ [35.4–45.9]	10.4 ^B^ [8.02–12.7]	6.22 ^C^ [5.56–6.88]
**Factory**	**pH**	**Water Activity (a_w_)**	**Moisture (%)**	**Mesophiles** **(log CFU/g)**
BRA1	5.46 ^C^ [5.40–5.52]	0.898 ^C^ [0.882–0.913]	37.7 ^C^ [35.1–40.2]	6.56 ^C^ [6.21–6.90]
BRA2	6.11 ^A^ [6.05–6.17]	0.860 ^D^ [0.844–0.876]	25.6 ^E^ [23.1–28.2]	6.58 ^C^ [6.23–6.92]
CAR	4.87 ^F^ [4.81–4.93]	0.910 ^BC^ [0.895–0.926]	38.2 ^C^ [35.6–40.7]	7.09 ^BC^ [6.74–7.43]
DOU	5.99 ^B^ [5.93–6.05]	0.851 ^D^ [0.835–0.866]	26.7 ^E^ [24.1–29.2]	7.22 ^B^ [6.87–7.56]
MIR1	4.97 ^F^ [4.91–5.03]	0.965 ^A^ [0.950–0.981]	43.0 ^B^ [40.5–45.6]	7.67 ^B^ [7.33–8.02]
MIR2	5.19 ^D^ [5.14–5.25]	0.934 ^B^ [0.918–0.950]	31.2 ^D^ [28.7–33.8]	7.33 ^B^ [7.00–7.68]
MIR3	4.98 ^F^ [4.92–5.04]	0.916 ^B^ [0.900–0.931]	39.0 ^BC^ [36.5–41.6]	5.61 ^D^ [5.27–5.96]
MIR4	5.21 ^D^ [5.15–5.27]	0.925 ^B^ [0.909–0.941]	39.2 ^BC^ [36.6–41.7]	7.31 ^B^ [6.97–7.65]
MOG	5.10 ^E^ [5.04–5.16]	0.948 ^AB^ [0.932–0.964]	48.5 ^A^ [46.0–51.1]	6.73 ^C^ [6.38–7.07]
VF	4.92 ^F^ [4.86–4.98]	0.910 ^BC^ [0.894–0.926]	40.1 ^BC^ [37.6–42.7]	8.68 ^A^ [8.33–9.02]
VIM	5.93 ^B^ [5.87–6.00]	0.803 ^E^ [0.788–0.819]	23.7 ^E^ [21.1–26.2]	6.58 ^C^ [6.24–6.93]
VIN1	5.50 ^C^ [5.45–5.56]	0.857 ^D^ [0.842–0.873]	21.5 ^F^ [19.0–24.1]	6.72 ^C^ [6.38–7.06]
VIN2	4.98 ^F^ [4.92–5.04]	0.882 ^C^ [0.866–0.898]	24.4 ^E^ [21.9–27.0]	7.12 ^BC^ [6.77–7.46]
VIN3	5.43 ^C^ [5.37–5.49]	0.805 ^E^ [0.789–0.821]	19.5 ^F^ [17.0–22.1]	6.45 ^C^ [6.10–6.79]
**Factory**	***S. aureus*** **(log CFU/g)**	***Clostridium* spp.** **(log CFU/g)**	**LAB on MRS** **(log CFU/g)**	**LAB on M17** **(log CFU/g)**
BRA1	2.02 ^A^ [1.59–2.45]	0.699 ^B^ [0.482–0.916]	8.93 ^B^ [8.50–9.35]	8.80 ^B^ [8.42–9.18]
BRA2	2.23 ^B^ [1.80–2.66]	1.395 ^A^ [1.178–1.611]	7.76 ^C^ [7.34–8.18]	7.86 ^C^ [7.48–8.24]
CAR	1.70 ^A^ [1.27–2.13]	0.699 ^B^ [0.482–0.916]	8.21 ^C^ [7.79–8.63]	8.04 ^C^ [7.66–8.42]
DOU	1.96 ^A^ [1.53–2.39]	0.759 ^B^ [0.543–0.976]	9.88 ^A^ [9.45–10.3]	9.44 ^B^ [9.05–9.82]
MIR1	2.38 ^B^ [1.94–2.81]	0.699 ^B^ [0.482–0.916]	9.30 ^B^ [8.88–9.73]	9.48 ^B^ [9.10–9.86]
MIR2	1.85 ^A^ [1.42–2.29]	1.848 ^A^ [1.631–2.064]	8.80 ^B^ [8.38–9.22]	8.59 ^B^ [8.20–8.97]
MIR3	1.70 ^A^ [1.27–2.13]	0.699 ^B^ [0.482–0.916]	9.14 ^B^ [8.72–9.56]	8.84 ^B^ [8.46–9.22]
MIR4	2.22 ^B^ [1.79–2.65]	0.699 ^B^ [0.482–0.916]	9.35 ^B^ [8.93–9.78]	9.22 ^B^ [8.84–9.60]
MOG	2.16 ^B^ [1.73–2.60]	0.699 ^B^ [0.482–0.916]	8.47 ^C^ [8.05–8.89]	7.66 ^C^ [7.28–8.04]
VF	1.76 ^A^ [1.33–2.19]	0.699 ^B^ [0.482–0.916]	10.2 ^A^ [9.79–10.6]	10.0 ^A^ [9.67–10.4]
VIM	1.70 ^A^ [1.27–2.13]	0.699 ^B^ [0.482–0.916]	9.20 ^B^ [8.78–9.62]	9.68 ^A^ [9.30–10.1]
VIN1	2.55 ^B^ [2.12–2.98]	0.759 ^B^ [0.543–0.976]	9.25 ^B^ [8.83–9.67]	9.12 ^B^ [8.74–9.51]
VIN2	1.82 ^A^ [1.39–2.25]	1.230 ^A^ [1.014–1.447]	9.12 ^B^ [8.70–9.54]	8.97 ^B^ [8.59–9.35]
VIN3	1.70 ^A^ [1.27–2.13]	0.759 ^B^ [0.543–0.976]	9.12 ^B^ [8.70–9.54]	8.93 ^B^ [8.55–9.31]

db: dry basis; Factories: BRA 1-2: Bragança 1-2; CAR: Carrazeda de Ansiães; DOU: Miranda do Douro; MIR 1-4: Mirandela 1-4; MOG: Mogadouro; VF: Vila Flor; VIM: Vimioso; VIN 1-3: Vinhais 1-3.

**Table 2:** Mean values of the main quality characteristics underpinning the three-cluster solution.

Quality Characteristic	Cluster 1 (Lowest pH, High Water Content, More Protein)	Cluster 2 (Higher pH, Low Water Content, More Fat)	Cluster 3 (Low pH, High Water Content, High Protein/Low Fat, High Ash, Improved Hygiene)
	Mean (±SD)	Mean (±SD)	Mean (±SD)
pH	5.04 (±0.12)	5.75 (±0.31)	5.10 (±0.24)
a_w_	0.922 (±0.03)	0.843 (±0.04)	0.918 (±0.02)
Moisture (%)	36.16 (±7.60)	23.82 (±4.00)	40.86 (±4.92)
Fat (% db)	34.83 (±10.91)	44.18 (±8.13)	30.94 (±5.93)
Protein (% db)	50.80 (±8.50)	41.62 (±6.87)	50.46 (±5.16)
Ash (% db)	5.78 (±1.56)	6.60 (±1.68)	9.19 (±1.00)
*S. aureus* (log CFU/g)	2.032 (±0.68)	2.002 (±0.46)	1.893 (±0.35)
*Clostridium* (log CFU/g)	0.927 (±0.43)	0.978 (±0.50)	0.699 (±0.00)
LAB on MRS (log CFU/g)	9.451 (±0.63)	8.984 (±0.82)	8.686 (±0.50)
LAB on M17 (log CFU/g)	9.382 (±0.54)	8.924 (±0.78)	8.337 (±0.61)

db: dry basis.

**Table 3:** Coefficients of correlation of the physicochemical and microbiological characteristics with the three varimax-rotated factors (Factors 1, 2, and 3) along with commonalities and explained variances.

Variable	Factor 1 Low pH, High Moisture	Factor 2 More Meat in Formulation	Factor 3 Longer or Rapid Fermentation	h2	u2	Commonalities
pH	−0.69	−0.08	−0.12	0.492	0.508	1.1
a_w_	0.99	−0.14	−0.05	0.981	0.019	1.0
Moisture	0.83	0.37	−0.06	0.819	0.181	1.4
Ash (db)	0.02	0.74	−0.33	0.664	0.336	1.4
Fat (db)	−0.32	−0.90	0.00	0.906	0.094	1.2
Protein (db)	0.36	0.76	0.17	0.730	0.270	1.6
Total mesophiles	0.32	−0.04	0.53	0.382	0.618	1.6
*S. aureus*	0.06	−0.27	−0.04	0.079	0.921	1.1
*Clostridium*	0.01	−0.38	−0.21	0.185	0.815	1.5
LAB on MRS	−0.01	0.14	0.93	0.886	0.114	1.0
LAB on M17	−0.13	0.07	0.96	0.943	0.057	1.0
Proportion Variance	0.235	0.208	0.206			
Cumulative Variance	0.235	0.442	0.649			

db: dry basis.

## Data Availability

The original contributions presented in the study are included in the article, further inquiries can be directed to the corresponding author.
